# The Cheerful Sick Room

**Published:** 1910-04

**Authors:** 


A Monthly Review of Medicine and Surgery.

*EDITOR*

WILLIAM WARREN POTTER, M. D.

All communications, whether of a literary or business nature, books for review and xchanges, should be addressed to the editor, 238 Delaware Avenue, Buffalo, N.Y.

Vol. Lxv. APRIL, 1910. No. 9

### The Cheerful Sick Room

RUTH CAMERON, the clever writer of "The Evening Chit Chat" in the *Globe* and *Commercial Advertiser* (New York), discoursed upon the sick room and methods of making it cheerful, in the edition of that paper of March 18, 1910. The article contains many hints of value on the subject and, moreover, it is a topic of so much importance that we reproduce Miss Cameron's remarks in their entirety. Especially we are glad to do so because a woman can present the case from the most advantageous viewpoint, embracing many details that a man is quite likely to overlook.

It is doubtful if the paragraph relating to the habit of following the doctor out of the room for confidential talk in the hallway or on the stairs, always within sound of the voices, has ever been more importantly stated than by Miss Cameron. We invite careful attention to her entire article, which is full of interest to every physician.

I wonder if all of us realize of what tremendously great importance it is to have a cheerful, normal atmosphere in a sickroom.

We put great stress on shutting out the worries of everyday life, but too often we succeed in also shutting out its vitality and cheerfulness and creating a lifeless atmosphere that would be bad for a well person and is doubly so, of course, for the sick man.

"Having an Indian war dance in a patient's room couldn't be any worse for him than this white-flowers-lowered-voice-bated breath atmosphere that some people think it is necessary to create in a sickroom," a nurse who is at the top of her profession said to me once.

I was reminded of that yesterday when I went to see a young girl who has just made a record recovery from appendicitis, and she told me that the thing that did the most to get her well was just *her nurse's red kimona.*

When they started to operate on me I knew they didn't expect me to live, so I was feeling pretty bad," she said, "but when I came out of the ether I saw the nurse sitting there so comfortable and cheerful and homelike-looking in that red kimono, that it didn't seem to me she could think I was as sick as that, and it gave me so much confidence that I began to feel better right away."

It is pretty hard for a sick person to tell just how ill he is. Most of us don't get to the flat-on-our-backs-send-for-a-doctor stage until we feel about as miserable as we can imagine feeling, and whether we really are on the verge of death or just feel so, we have to tell by the action of those about us.

Wherefore it is distinctly up to those about sick folks to create as cheerful and unalarming an atmosphere as possible.

The habit of following the doctor out of the room and holding converse with him just out of the patient's earshot is an exceptionally vicious one. It doesn't make any difference if you simply ask the doctor, "Shall I keep the medicine in a cool place?" and he answers, "Yes."

The patient is perfectly sure that you asked if he were likely to live, and the doctor said he wasn't.

Never send white flowers to a sick person. They are altogether too suggestive. Even violets are a trifle too funereal Bright red and yellow flowers are the best. Sunshine, you know, is a great healer, and such flowers catch and hold the sunshine long after its direct rays have departed. Too many cut flowers are said to vitiate the air of a sickroom. Growing plants are a much better gift for a sick person. And if you buy a plant be sure to get one that has two or three buds. In the uneventful life of a sickroom the development of a flower is often an absorbing and delightful interest.

Never allow a lugubriously inclined person to see a patient, especially a nervous patient. I actually know of one case where a patient, ill with nervous trouble, had a distinct relapse when a kindly neighbor who was allowed to see her saluted with, "Why, how you have fallen away!"

And yet, while you should be careful not to let the patient see you think he is very ill, don't go to the other extreme of making light of his symptoms.

It isn't good for a patient to have preliminary funeral services held over him in the expression and attitude of his friends, but it doesn't hurt him to have a little kindly sympathy for his aches and pains.

The golden mean is as good a thing to follow in a sickroom as elsewhere, and the very best atmosphere one can create about a sick person is the atmosphere of perfectly normal everyday life and cheerfulness.

